# Chronic Cardiac Conduit Masses in an Adolescent With a Right Ventricle-to-Pulmonary Artery Conduit

**DOI:** 10.1093/cid/ciag395

**Published:** 2026-10-28

**Authors:** Geraldine Huynh, Catherine Burton, Yashu Coe, Joan Robinson

**Affiliations:** Division of Pediatric Infectious Diseases, Department of Pediatrics, University of Alberta, Edmonton, Alberta, Canada; Division of Pediatric Infectious Diseases, Department of Pediatrics, University of Alberta, Edmonton, Alberta, Canada; Division of Pediatric Cardiology, Department of Pediatrics, University of Alberta, Edmonton, Alberta, Canada; Division of Pediatric Infectious Diseases, Department of Pediatrics, University of Alberta, Edmonton, Alberta, Canada

## QUESTION

A fully immunized 17-year-old girl from a remote Canadian Indigenous community was admitted for evaluation of masses in her right ventricle-to-pulmonary artery (RV-PA) conduit. Tetralogy of Fallot was repaired at 12 months of age with an RV-PA homograft conduit, followed by multiple PA balloon dilatations and a left PA stent, last dilated 6 years prior.

Seventeen months prior to diagnosis, echocardiography that led to a computed tomography (CT) chest scan showed twenty 2–6-mm masses within the conduit and scattered ground-glass and cavitary pulmonary nodules (Figure 1, Supplementary Figure 2). The patient was afebrile with no respiratory symptoms. Three blood cultures had no growth at 5 days. Interferon-gamma release assay was positive. *Histoplasma* and *Blastomyces* serology, serum galactomannan, and human immunodeficiency virus (HIV) serology were negative. Bronchoalveolar lavage (BAL) and endobronchial ultrasound-guided biopsy of the lung and a lymph node were performed. Her BAL grew *Streptococcus pneumoniae* and *Haemophilus influenzae*, treated with 5 days of amoxicillin. Her mycobacterial, fungal cultures, and tuberculosis (TB) polymerase chain reaction (PCR) were negative from the BAL and biopsies. Pathological examination of the lung and lymph node showed no malignancy or granulomas. Once mycobacterial cultures were confirmed negative, the patient received 12 weeks of isoniazid and rifapentine for latent TB infection. A CT chest scan then showed resolution of the cavitary lesions but new ground-glass nodules.

**Figure 1.**
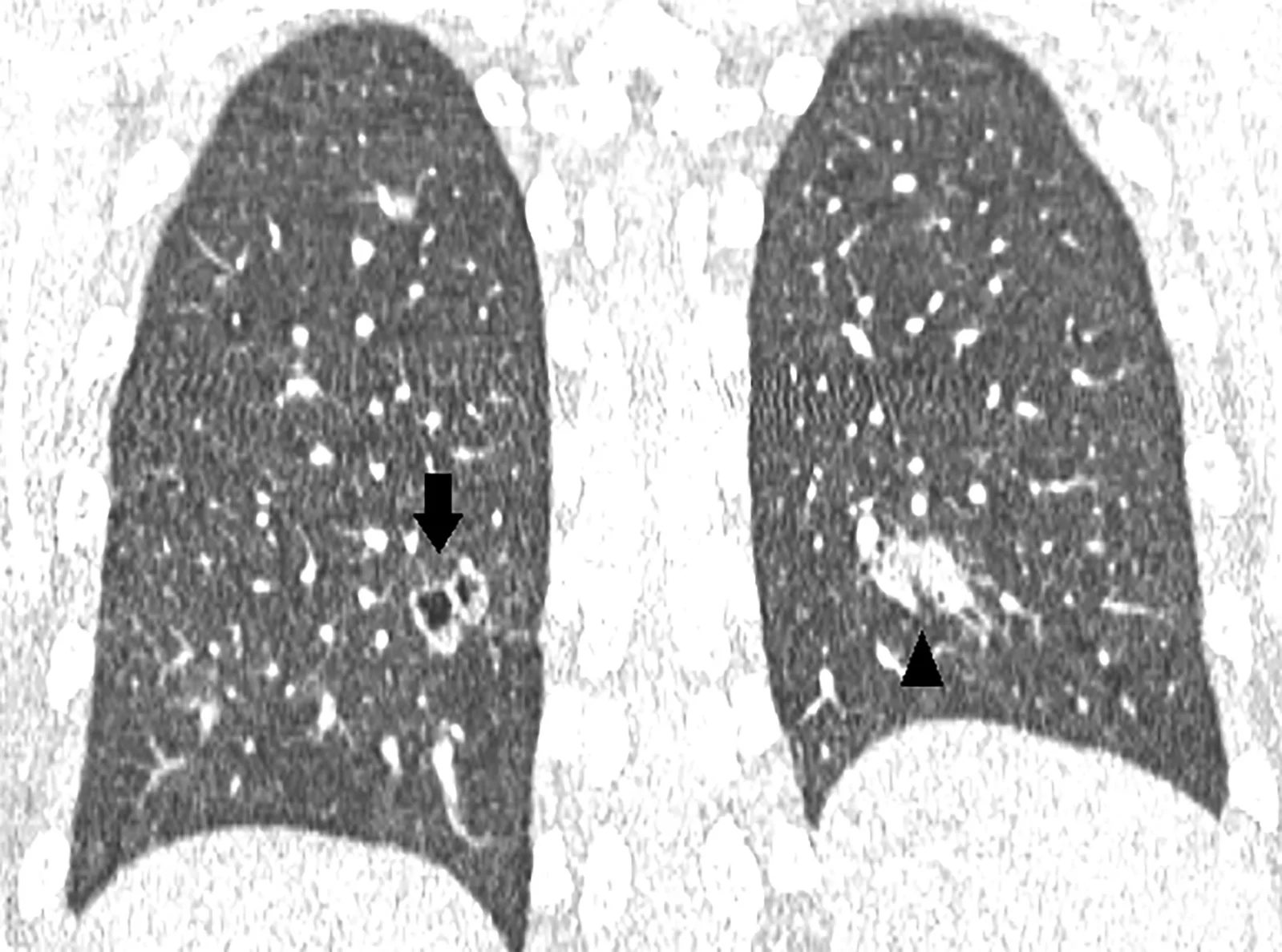
Coronal computed tomography lung image showing cavitary lesions in the right lower lobe (black arrow) and peribronchovascular consolidation in the left lower lobe (black arrowhead), in keeping with septic emboli.

One month before admission, a routine echocardiogram showed new masses in the conduit and on the tricuspid leaflet (Figure 2, Supplementary Figure 1). Chest and upper abdominal CT demonstrated splenomegaly, extension of the conduit lesions into the PA stent, and almost complete resolution of pulmonary disease. She also had a 9-month history of nonblanchable palpable purpura of the lower extremities (Figure 3). Investigations showed the following: C-reactive protein (CRP), 33.8 mg/L (normal, <8 mg/L); C3, 0.36 g/L (normal, 0.6–1.6 g/L); C4, 0.02 g/L (normal, 0.10–0.40 g/L); hematuria; urine protein/creatinine ratio, 155 mg/mmol (normal, <13 mg/mmol); hemoglobin (Hb), 86 g/L; and antinuclear antibody, 1:320.

**Figure 2.**
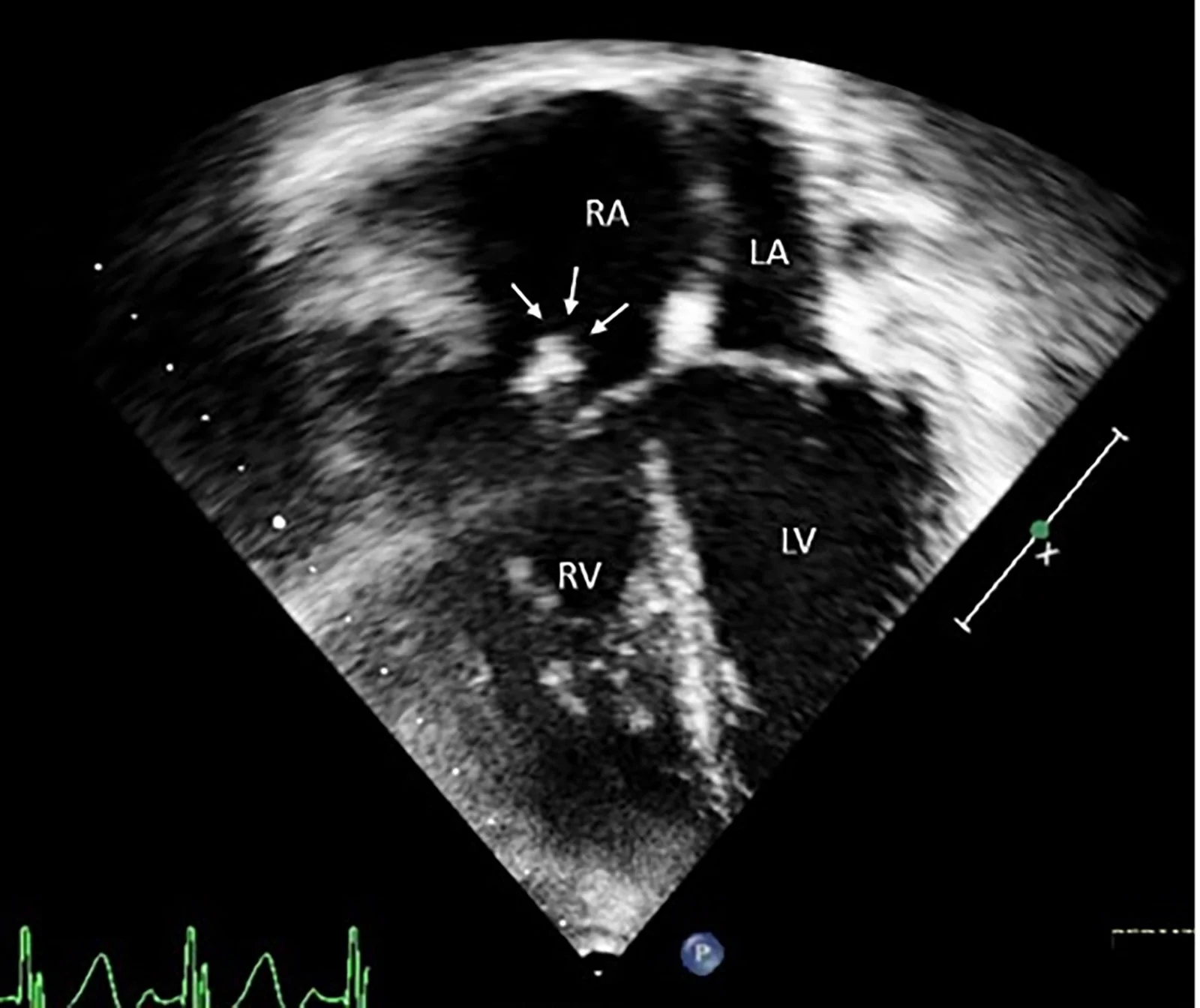
Echocardiography showing tricuspid valve vegetation, 12 × 5.3 mm (arrows). Abbreviations: LA, left atrium; LV, left ventricle; RA, right atrium; RV, right ventricle.

**Figure 3.**
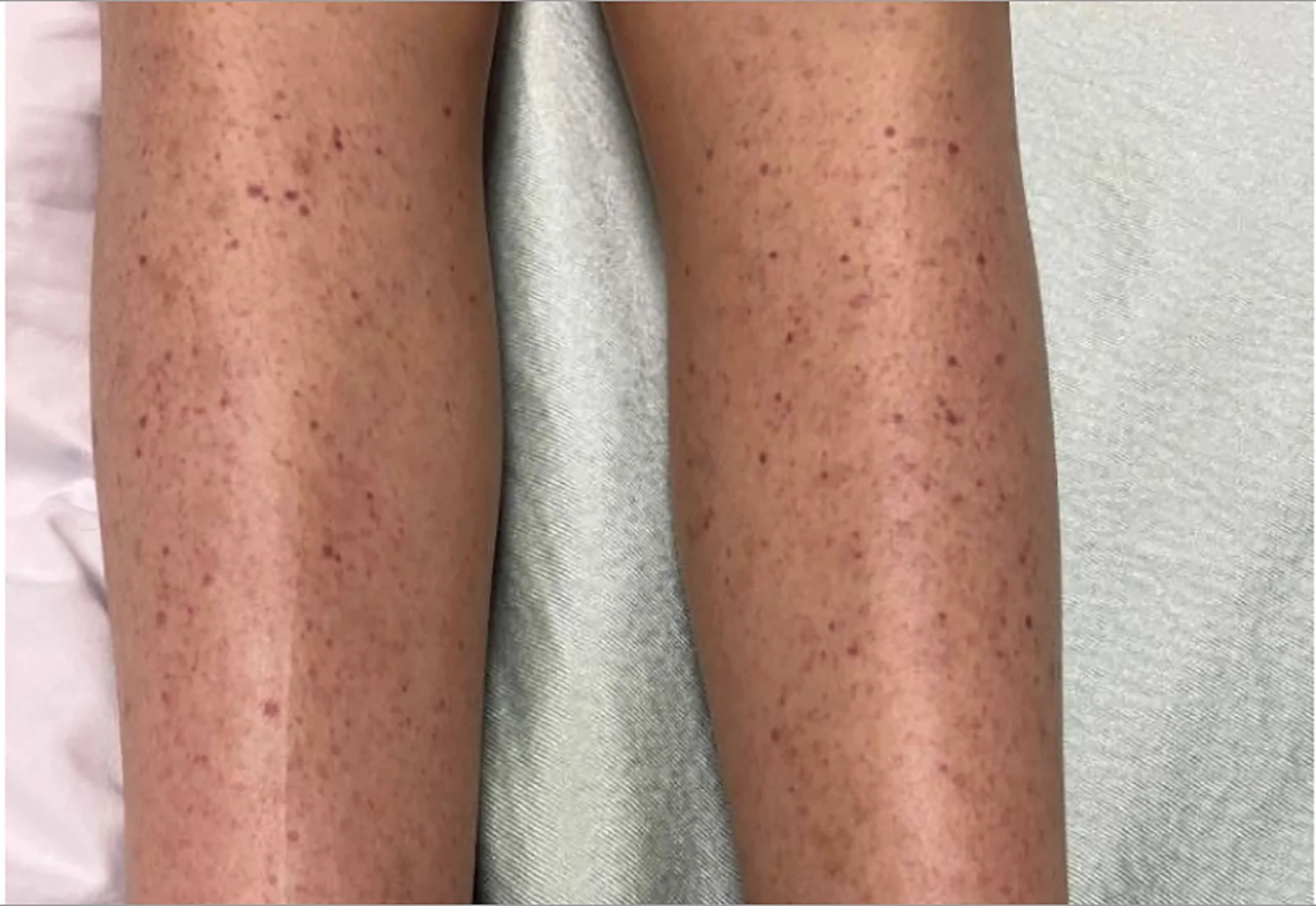
Nonblanchable palpable purpura of bilateral lower extremities.

The patient denied fevers but endorsed fatigue, depression, poor appetite, exercise intolerance (NY Heart Association [NYHA] class III), chills, headaches, intermittent ankle swelling, and alopecia. A diagnostic result was returned and prompted admission.

What is your diagnosis?

## ANSWER TO THE PHOTO QUIZ

Diagnosis: *Bartonella quintana* infective endocarditis (IE), confirmed by *Bartonella* serology (>1:8192) and blood and heart tissue nucleic acid amplification testing (NAAT) positive for *B quintana* and negative for *B henselae*

*Bartonella* spp is a small, fastidious gram-negative bacillus that causes approximately 2%–3% of IE in Canada [1, 2]. Four species of *Bartonella* cause IE: *B quintana*, *B henselae*, *B elizabethae*, and *B vinsonii* subsp *Berkhoffii* [2], with *B quintana* and *B henselae* being the most common. *B quintana* is an increasingly recognized cause of culture negative IE. A 2023 systematic review found 160 adults and 7 children with *B quintana* IE. Fifty-two (31%) had valvular disease, most commonly bicuspid aortic valves (n = 12). Embolization was documented in 43 cases (26%), most commonly to the spleen. Death occurred only in adults (n = 16 [10%]) [3]. A 2025 report added a novel pediatric case linked to head lice [4].

A hallmark of *B quintana* IE is its chronicity due to slow replication [5]. The organism lives within red blood cells, allowing prolonged bacteremia and immune activation that can lead to embolic and multisystem complications [3]. Infection typically evolves over months to years with nonspecific symptoms, including fatigue, anemia, splenomegaly, renal disease, and other immune-mediated phenomena. Our patient had all of these findings plus a vasculitic-appearing rash (Figure 3). To our knowledge, bacillary angiomatosis has not been described with *B quintana* IE [3] and biopsy of the patient’s rash showed nonspecific perivascular lymphocytic dermatitis. Fever is often absent, as in our case [3].

The pulmonary findings were diagnostically challenging and resolved prior to *Bartonella*-specific therapy. We did not prove microbiologically that they were *Bartonella*-related septic emboli. However, their distribution, cavitary/ground-glass morphology, her chronic right-sided endocarditis, and the absence of an alternative primary pulmonary process made septic pulmonary emboli the favored explanation.

*B quintana* is classically associated with being unhoused, poor hygiene, poverty, alcohol dependence, overcrowding, and body lice [4–6]. However, emerging data demonstrate that head lice can also act as a vector [4, 5] and lack of access to clean water for bathing is a risk factor, particularly in pediatric populations [3, 4]. After diagnosis, further history revealed recurrent household head lice infestations and a likely history of head lice in the patient herself, which appeared to be the most compelling epidemiologic exposure. She also reported adult cat scratches, but *B henselae* testing was negative. The absence of body lice or being unhoused highlights that epidemiologic risk of *B quintana* extends beyond traditional exposures, particularly among children and adolescents [2, 5].

Blood cultures are typically negative in *Bartonella* IE, even with prolonged incubation. *Bartonella* serology is the key to diagnosis; immunoglobulin G (IgG) titers ≥1:800 is a major Duke criterion [3]. Speciation requires molecular testing, which is sometimes falsely negative with chronic disease [7] but in our case detected *B quintana* from blood and heart tissue; 16S rRNA appears to be more sensitive than PCR [3, 4] when performed on blood. PCR testing of excised valvular tissue is the diagnostic standard for IE [5, 8].

Expert opinion is that optimal therapy for *B quintana* IE consists of doxycycline for at least 6 weeks, combined with gentamicin or rifampin for synergy [3, 6, 9]. The patient was started on oral doxycycline and rifampin and underwent RV-PA conduit replacement and aortic and tricuspid vegetectomies 11 days later. Although the patient received rifapentine to treat her latent TB infection, the role of this exposure in the interval improvement of the pulmonary lesions is uncertain, especially as it did not prevent progression of intracardiac disease and is not adequate treatment alone for *Bartonella* IE.

In a systematic review, 154 of 167 (98%) of cases of *B quintana* IE had cardiac surgery, likely reflecting delayed diagnosis, destructive intracardiac disease, and embolic risk [5]. It is unclear whether postponement of surgery until antibiotics have been administered for days to weeks is of benefit or increases the risk of emboli.

Whole-body positron emission tomography (PET), pan-CT imaging, and ultrasound identified the heart as the primary site of infection, with septic emboli to the lungs and a renal artery pseudoaneurysm but no evidence of intracranial embolic disease. The patient’s renal artery pseudoaneurysm was embolized 2 weeks postsurgery with no complications. She completed 12 weeks of doxycycline with rifampin for the first 6 weeks. The patient's moderate left ventricular dysfunction resolved and she had significant clinical improvement (NYHA class I) after surgery and antibiotic therapy.

This case illustrates the chronicity and immune-mediated manifestations of *B quintana* IE. *B quintana* should be considered in patients with culture-negative endovascular infection and nontraditional exposures, including head lice. Diagnosis relies on serology and molecular testing. Disease is often indolent, yet destructive, frequently requiring prolonged combination antimicrobial therapy and cardiac surgery, underscoring the importance of early recognition to prevent severe complications.

## Supplementary Material

ciag395_Supplementary_Data

## Supplementary Data

Supplementary materials are available at *Clinical Infectious Diseases* online. Consisting of data provided by the authors to benefit the reader, the posted materials are not copyedited and are the sole responsibility of the authors, so questions or comments should be addressed to the corresponding author.
