# Supplementary material for: Chronic Cardiac Conduit Masses in an Adolescent With a Right Ventricle-to-Pulmonary Artery Conduit
Source: Clin Infect Dis. 2026 Oct 28;83(4):e900–3. doi: 10.1093/cid/ciag395 (PMC13715962; doi:10.1093/cid/ciag395)
Supplement: Supplementary file 1 [file ciag395_supplementary_data.zip › supplementary figure legends.docx]

Supplementary Figure Legends

**Supplementary Figure 1:** Echocardiography showing multiple masses in Right ventricule-to-Pulmonary Artery conduit (solid white arrows) and right pulmonary artery (solid red arrows)

**Supplementary Figure 2:** Contrast-enhanced Computed Tomography sagittal image of the Right Ventricle-to-Pulmonary Artery conduit shows calcified severe narrowing at the proximal anastomosis of the conduit (black arrow) with post-stenotic dilation. Peripheral frond-like filling defects (black arrowhead) within the conduit correspond to thrombi.
